# Segment assembly, structure alignment and iterative simulation in protein structure prediction

**DOI:** 10.1186/1741-7007-11-44

**Published:** 2013-04-15

**Authors:** Yang Zhang, Jeffrey Skolnick

**Affiliations:** 1 Department of Computational Medicine and Bioinformatics, University of Michigan, Ann Arbor, MI 48109, USA; 2 Center for the Study of Systems Biology, School of Biology, Georgia Institute of Technology, Atlanta, GA 30318, USA

## 

It has been 50 years since Anfinsen first showed that the native structure of protein molecules is determined solely by their amino acid sequence, with the folded state representing a unique and kinetically accessible minimum of the free energy [1]. This finding, also known as the Anfinsen's thermodynamic hypothesis, motivated the belief that the solution to the protein structure prediction problem should be based on physicochemical principles; that is, all one has to do to find the native structure of a protein is to identify the lowest free-energy state. However, the success of such first principle-based methods has been modest at best. Knowledge-based approaches, which predict structural models using the regularities and rules of known protein structures seen in the Protein Data Bank (PDB) library, have enjoyed more extensive success in protein structure prediction [2-4]. Among these approaches, TASSER (Threading ASSEmbly Refinement) is a hierarchical structure modeling method designed to predict full-length atomic models from primary amino acid sequences [4]. For a given query sequence, TASSER first threads the sequence through the PDB to identify template proteins that may have similar topology to the query. Continuous segments are then excised from the top-ranked template structures following the query-template alignments, and these are reassembled into full-length atomic models by Monte Carlo simulations. An essential advantage of TASSER over traditional comparative modeling methods, which often deteriorate the quality of template models, is its ability to drive the template structures closer to the native than the input templates. This is mainly attributed to its highly optimized knowledge-based potential and the efficiency in combining complementary threading alignments from multiple template structures.

The I-TASSER (Iterative Threading ASSEmbly Refinement) method that we published in *BMC Biology *in 2007 is an extension of the TASSER algorithm for iterative structure assembly and refinement of protein molecules [5]. The idea of I-TASSER was inspired by the encouraging template structure refinement of the TASSER simulations. Here, a highly appealing question is to examine whether we can continuously improve the quality of protein structural models by repeatedly re-folding the query sequence starting from the last step of assembly simulations. The result of initial tests was not encouraging since the final structural models stay essentially the same as the first round of TASSER models, although the local structural quality, including hydrogen-bonding networks and steric clash of backbone atoms, was generally improved. The reason for the failure in structural refinement became obvious once it was realized that no new structural information was introduced in the reassembly simulations by simply starting from the last round TASSER models. As long as the dynamic searching of conformational space is complete in the first round of simulations, the iterations should in principle lead to exactly the same modeling results. More pronounced topology-level improvements were achieved when new structural templates identified from the PDB were incorporated into the folding iterations; these templates were detected by the structure alignment program TM-align [6], which matches the TASSER models with each of the known proteins in the PDB to identify the templates that are structurally closest to the TASSER models. In a benchmark testing experiment on a set of known proteins, the TM-score of the template structures identified by TM-align (a measure of similarity between model and native with value in 0[1]) is shown to be generally lower than that of the TASSER models (only 21% of the TM-align alignments have a higher TM-score, as seen in Figure 4A of [6]). Nevertheless, the combination of the new structural alignment information in the I-TASSER simulations eventually resulted in final models with improved TM-score in 77% of the test proteins, or an overall TM-score increase of approximately 3% with improved local structure quality [5].

The idea of model assembly iterations driven by structure alignments has recently found promising uses in both atomic-level structure refinement and *ab initio *protein folding [7,8]. Starting from low-resolution models generated by coarse-grained structure modeling or low-resolution experiments, the goal of structural refinement is to improve the quality of the structural models by driving the structure closer to the native. In the development of FG-MD (Fragment Guided-Molecular Dynamics simulation) [7], a method for atomic-level structural refinements, the initial models to be refined are split into segments spanning two to four secondary structure elements; template segment structures similar to that split from the initial models are then identified from the PDB library by TM-align. The atomic contact and distance maps collected from the segmental templates, which often have an improved local geometry over the initial models, can reshape the energy distribution of the physics-based molecular dynamics simulations [9], where a funnel-like shape of the energy landscape with the native at the bottom is often required for efficient structural refinements. Under the constraints of the fragment-based contact and distance maps, the molecular dynamics simulations resulted in consistent atomic-level refinement for the majority of structural models that have the correct topology with a TM-score >0.5 (Figure 1).

**Figure 1:**
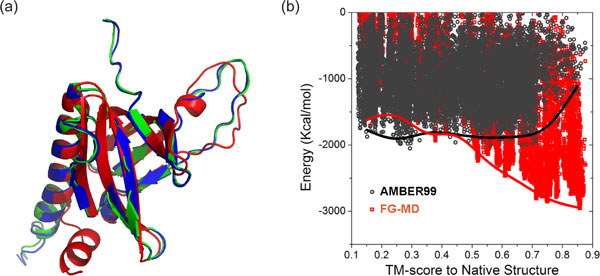
**An example of structure refinement by FG-MD (Fragment-Guided Molecular Dynamics simulation) for TR614, the pleckstrin homology domain of *Saccharomyces cerevisiae *Avo1, a structure refinement target in CASP9 (the 9^th ^Critical Assessment of protein Structure Prediction experiment)**. The initial structure is among the best models selected by the organizer from the CASP9 server predictions. Segment structures searched by TM-align were used to reshape the MD energy landscape, which resulted in a TM-score increase by 9 units. **(a) **Superposition of the initial model (green), the refined model (blue) and the native structure (red). **(b) **A plot of energy versus TM-score for a set of 77×220 refined models generated by the conventional molecular dynamic simulation program AMBER99 (black circles) and FG-MD (red squares), respectively, starting from 77 decoy models of different resolutions. The curves are connection of the medians of the ten lowest-energy models in each of the TM-score bins, where a funnel-like shape appears in the energy landscape of the FG-MD simulations but is absent from that of the AMBER99 simulations.

As the success of structure assembly iterations relies on the new structural information introduced in the simulations, more efficient structural improvements were achieved when using the models built by *ab initio *folding as the probe to detect new templates. Since the probe models were constructed from scratch, any reasonable match of the *ab initio *models with real experimental proteins is significant and often indicates correct folding template identifications. Figure 2 shows two such examples achieved in the recent 10^th ^CASP (Critical Assessment of protein Structure Prediction) experiment, a community-wide experiment designed to assess computational methods with all modeling predictions generated before the experimental structures are released [10]. In these examples, low-resolution models were first built from the query sequence by QUARK, an *ab initio *protein folding algorithm that assembles structural models from random conformations as guided by a composite physics- and knowledge-based force field [8]. Based on the QUARK models, structural templates with an increased TM-score were fished out by TM-align from the PDB. Final models with correct folds (TM-score >0.5) were obtained by further I-TASSER refinements for proteins of more than 150 residues, an unprecedented success in terms of the size of the successfully folded proteins in Free Modeling in the CASP experiment.

**Figure 2:**
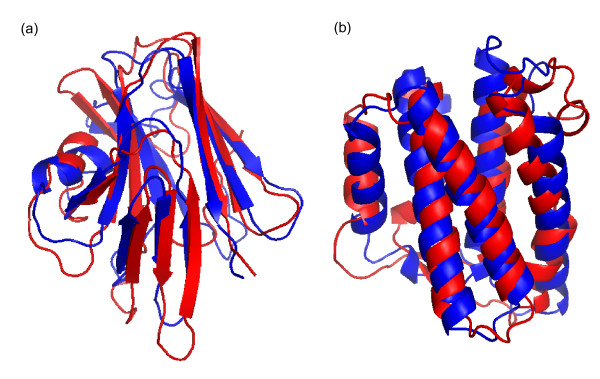
**Successful modeling of two Free-Modeling (FM) targets by Zhang-Server in CASP10.** Blue and red are predicted model and experimental structure, respectively. **(a) **R0006 (a protein encoded in the genome of *Bacteroides thetaiotaomicron*, VPI-5482) is a beta protein of 169 residues. The *ab initio *folding algorithm QUARK generates five models of the highest TM-score = 0.32; based on the QUARK models, the TM-align search identifies a template of TM-score = 0.5, which results in the final submitted model with a TM-score = 0.622 after the I-TASSER refinements. **(b) **R0007 (the human interleukin-34 protein) is an alpha protein with 161 residues. QUARK generates models of the highest TM-score = 0.43; based on the QUARK models the TM-align search identifies a template of TM-score = 0.48, which results in a final model of TM-score = 0.620 after the I-TASSER refinements.

The major goal of protein structure prediction is to help understand the biological roles of protein molecules in living cells. Since the launch, at the beginning of this century, of structural genomics projects that aim to solve experimentally the structure of a set of proteins covering all representative structural types in nature [11], the general challenges to the field of structure prediction have been the development of methods for better template identification and consequent structural refinement. Progress has been substantial but significant difficulties still remain in distant-homology identifications and atomic-level structure refinements despite the fact that the PDB library is approaching completeness in structural space [12,13]. Meanwhile, it is now known that nearly 10% of all proteins (or partial sequence in 40% of eukaryotic proteins) do not follow Anfinsen's dogma to fold into unique states - these sequences are unfolded or intrinsically disordered to conduct their physiological functions [14]. Template-based approaches cannot be used for deducing structural and functional characteristics of these molecules. While recent progress has demonstrated promising use of the structure-based iteration as driven by *ab initio *modeling in both fold-recognition and structure refinement procedures, the development of efficient *ab initio *folding algorithms will remain a major theme in the field and should have important impacts on all aspects of protein structure predictions.

## Note

This article is part of the BMC Biology tenth anniversary series. Other articles in this series can be found at http://www.biomedcentral.com/bmcbiol/series/tenthanniversary.
